# Deletion of the sex-determining gene *SXI1α* enhances the spread of mitochondrial introns in *Cryptococcus neoformans*

**DOI:** 10.1186/s13100-018-0129-0

**Published:** 2018-07-17

**Authors:** Zhun Yan, Zhimin Li, Li Yan, Yongting Yu, Yi Cheng, Jia Chen, Yunyun Liu, Chunsheng Gao, Liangbin Zeng, Xiangping Sun, Litao Guo, Jianping Xu

**Affiliations:** 1 Institute of Bast Fiber Crops, Chinese Academy of Agricultural Sciences, Changsha, 410205 Hunan China; 2 Department of Biology, McMaster University, Hamilton, ON L8S 4K1 Canada

**Keywords:** HEG, Intron, Mating type, mtDNA inheritance

## Abstract

**Background:**

Homing endonuclease genes (HEGs) are widely distributed genetic elements in the mitochondrial genomes of a diversity of eukaryotes. Due to their ability to self-propagate within and between genomes, these elements can spread rapidly in populations. Whether and how such elements are controlled in genomes remains largely unknown.

**Results:**

Here we report that the HEG-containing introns in the mitochondrial *COX1* gene in *Cryptococcus neoformans* are mobile and that their spread in sexual crosses is influenced by mating type (*MAT*) α-specific homeodomain gene *SXI1*α. *C. neoformans* has two mating types, *MAT***a** and *MAT*α***.*** In typical crosses between strains of the two mating types**,** only a small portion (< 7%) of diploid fusants inherited the HEGs from the *MAT*α parent. However, disruption of the *SXI1*α gene resulted in the majority (> 95%) of the diploid fusants inheriting the HEG-containing introns from the *MAT*α parent, a frequency significantly higher than those of intronless mitochondrial genes.

**Conclusions:**

Our results suggest that *SXI1*α not only determines uniparental mitochondrial inheritance but also inhibits the spread of HEG-containing introns in the mitochondrial genome in *C. neoformans*.

## Background

Homing endonuclease genes (HEGs) are mobile genetic elements that can spread from HEG-containing (HEG^+^) alleles to their cognate alleles without HEG (HEG^−^). HEGs are typically located in introns and are broadly distributed in all three Domains of life, including in both the nuclear and organelle genomes of all major groups of eukaryotes [1]. However, despite their broad distribution, HEGs are not known to have any positive contribution to host fitness and function. Rather, their sole purpose seems to be enhancing their own spread. Consequently, HEGs have traditionally been considered selfish or parasitic genetic elements [2, 3]. The rapid spread of HEGs is achieved by a process termed “homing” which involves recognition and cutting of the HEG^−^ allele by the HEG-encoded endonuclease. The disrupted HEG^−^ allele is then repaired via a recombination-dependent repair system using an intact HEG^+^ allele as template. In contrast, the HEG^+^ allele is immune to such a cleavage because the presence of HEG interrupts the recognition and cleavage site. The end result of intron homing is the insertion of HEG and its associated intron into the HEG^−^ locus, leading to the presence of HEGs on both alleles [4, 5].

The high efficiency of the “homing” process in nature can be seen from the distribution patterns of HEGs in both natural populations as well as in laboratory genetic crosses [6, 7]. In sexual crosses, HEGs are often inherited by more than 95% of progeny rather than the typical Mendelian proportion of 50% for nuclear alleles [7, 8]. With such a high transmission frequency, a HEG gene could increase its frequency from 0.001 to 0.999% in about 15 outcrossed generations in a sexual population [3]. Indeed, in a survey of the ω-HEG and associated group I intron in the mitochondrial genomes of 20 ascomycete yeast species, Goddard et al. (1999) showed that the distribution of ω-HEG was not clustered on the host phylogeny and that the phylogeny based on ω-HEG sequences differed significantly from that of the host species as inferred using house-keeping genes. Their analyses suggested a cyclical model of ω-HEG invasion, degeneration, and loss, followed by reinvasion etc. [3].

While HEGs are prevalent in the mitochondrial genomes of many fungal species, they are noticeably absent in the mitochondrial genomes of animals [9]. One major difference between the fungal and animal mitochondrial genomes is their modes of transmission in sexual crosses. In animals, the mitochondrial genome is inherited almost exclusively from the maternal parent [9–11]. However, in fungi, there is a diversity of patterns, ranging from strictly uniparental to biparental [12, 13]. In addition, in an increasing number of fungal species, there are significant polymorphisms in HEG distributions among individuals within populations [14–16]. Such distribution patterns suggest that there are likely genetic mechanisms controlling the transmission of HEGs.

One hypothesis suggested that uniparental organelle inheritance might have evolved to control the spread of selfish elements in organelle genomes [10, 17–19]. Consistent with this hypothesis, in the plant fungal pathogen *Ustilago maydis*, uniparental mitochondrial inheritance was associated with a lower transmission efficiency of HEGs in the mitochondrial genome [20, 21]. However, HEGs in the chloroplast genome of the algae *Chlamydomonas* are freely transferred to the sexual zygotes despite the uniparental inheritance of the chloroplast genome [22, 23]. At present, the reasons for the different results between the two different organelles and species remain unknown.

In the basidiomycete yeast *Cryptococcus neoformans* species complex (CNSC)*,* sexual mating is controlled by one locus with two mating types, mating type **a** (*MAT***a**) and mating type α (*MAT*α). In typical crosses between strains of *MAT***a** and *MAT*α, the progeny inherit their mitochondrial genome almost exclusively from the *MAT***a** parent [24]. We previously identified that a *MAT*α specific gene, *SXI1*α, plays an important role in controlling mitochondrial inheritance in CNSC. Disruption of this gene resulted in biparental mitochondrial inheritance, significant heteroplasmy, and the recovery of recombinant mitochondrial genomes [25]. Furthermore, studies have shown that the mitochondrial genomes of this species complex are small (~ 24 to 33 kb) and polymorphic in the distribution of HEGs and their associated introns [14, 26]. However, the potential role of *SXI1*α in the spread of mitochondrial HEGs has not been investigated. The small mitochondrial genome size, the naturally existing HEG distribution polymorphisms in mitochondrial genomes among strains, and the presence of a gene that is known to control uniparental mitochondrial inheritance present a unique opportunity to investigate the potential mechanism of mitochondrial HEG mobility in CNSC.

## Methods

### Strains

The strains used for crossing experiments are listed in Table 1. These strains are all serotype D (*Cryptococcus neoformans* var. *neoformans*) and are isogenic except at the indicated loci. The origins of strain YZX2 and of strains CHY618, CHY620, CHY647 and CHY648 were described in Yan et al. [25] and in Hull et al. [27] respectively. Briefly, YZX2 is a *MAT***a** strain with a mitochondrial genotype characteristic of serotype A strains (*Cryptococcus neoformans* var. *grubii*) at the NADH dehydrogenase subunit #2 and #4 (*ND2* and *ND4*) loci [25]. The mitochondrial genome of the representative serotype A strain H99 is 24,874 bp long and contains only one intron, located in the cytochrome b (*COB1)* gene (GenBank accession number NC004336). Strain YZX2 is auxotrophic for adenine and contains a neomycin phosphotransferase gene (*NEO*) coding for G418 (geneticin) resistance [25]. In contrast, strains CHY618, CHY620, CHY647 and CHY648 were derived from the serotype D, *MAT*α strain JEC21 and they are all expected to have a mitochondrial genome, including intron distribution, identical to that of strain JEC21. While the complete mitochondrial genome of JEC21 is not available in public databases, it has been analyzed and reported as 33,194 bp long and contains the same number of introns at the same positions as that of strain IFM5844 [14, 26]. IFM5844 is a serotype D strain with deposited mitochondrial genome sequence through multiple GenBank accessions (AY138989, AF534132, AF534567, AF538354, AF538355, AF532780, AY560607, AY560609, and AY560611) [14]. Specifically, both strains IFM5844 and JEC21 share a total of 10 mitochondrial introns dispersed in four genes: two each in *COB1* and the large subunit of the ribosomal rRNA (*LsrRNA)* gene, five in the cytochrome c oxidase I (*COX1)*, and one in NADH dehydrogenase subunit 5 (*ND5*) [14, 26]. Sequence analysis of the mitochondrial genomes of strains JEC21 and IFM5844 revealed that the two introns in the *COB1* gene and four of the five introns in the *COX1* gene contained the LAGLIDADG motif, characteristic of HEGs [14, 26]. These well-characterized mitochondrial features make CNSC an excellent model from which to investigate the potential mobility of mitochondrial HEGs and their associated introns.

**Table 1:** Parental strains used in this study and their genotypes

Strain	Genotype^a^	Reference
CHY618	*MAT*α *ura5* mtD *sxi1*α::*NAT*	Hull et al. 2002
CHY620	*MAT*α *ura5* mtD *SXI1*α *NAT*	Hull et al. 2002
CHY647	*MAT*α *ura5* mtD *sxi1*α::*NAT URA5 ectopic pPM8 vector*	Hull et al. 2002
CHY648	*MAT*α *ura5* mtD *sxi1*α::*NAT URA5 ectopic pPM8-SXI1*α	Hull et al. 2002
YZX2	*MAT***a** *ade2* mtA *NEO*	Yan et al. 2004

^a^The above five strains are isogenic except at the indicated loci. *MAT***a**: mating type **a**; *MAT*α: mating type α. Strains with *ura5* or *ade2* auxotrophic markers require uracil or adenine respectively for growth on the minimum SD medium. mtA and mtD refer to the two parental mitochondrial genotypes distinguished by polymorphisms at loci *ND2*, *ND4*, *ND5* and *COX1*. mtD contains one and four extra introns in *ND5* and *COX1* respectively compared to mtA in strain YZX2. For details of alleles at these three loci as well as at the *COX1* locus, please see the main text, Fig. 1, Table 3, and references Xu (2002) and Yan and Xu (2003)

For strain CHY618, the sex-determining gene *SXI1*α was disrupted by the gene coding for *NAT* (nourseothricin) resistance. In contrast, strain CHY620 contains the wild-type *SXI1*α gene but with an ectopic copy of the *NAT* resistance gene [25, 27]. Thus, strain CHY620 serves as a reference control of CHY618 in sexual crosses. Strain CHY648 was derived from CHY618 where an ectopic copy of *SXI1*α in vector pPM8 was re-introduced into CHY618. Plasmid pPM8 is a *C. neoformans* shuttle vector encoding the *URA5* gene. It contained a BamHI site into which the *SXI1*α gene was cloned and then transformed into strain CHY618 to derive strain CHY648 [27]. Lastly, strain CHY647 is a negative control strain of CHY648 where the empty vector pPM8 without the ectopic copy of *SXI1*α was re-introduced into CHY618 [25, 27]. The genotypes of these parental strains are shown in Table 1.

### Mating and selection of diploid fusants

To prepare cells for mating, all five strains were first retrieved from the -80 °C freezer, spread onto YEPD (1% Bacto Yeast Extract, 2% Bacto Peptone, 2% dextrose, 2% agar) plates, and allowed to grow at room temperature for 2–4 days. Four pairs of strains (CHY618 x YZX2, CHY620 x YZX2, CHY647 x YZX2 and CHY648 x YZX2) were then mated on V8-jiuce medium [5% V8-vegetable juice (Campbell Soup Co.), 0.5 g/L KH_2_PO_4_, 4% agar and pH 7.2], following our previously described method [25]. After 16–20 h incubation, the mating mixtures were serially diluted and transferred to the minimum medium synthetic dextrose (SD) agar [1.7 g Yeast Nitrogen Base without Amino Acids (DIFCO), 20 g Dextrose, 5 g (NH_4_)_2_SO_4_, 20 g Agar, per liter] supplemented with both nourseothricin and geneticin (G418) to select for diploid fusants representing independent mating events. After 4 days of growth on the selective medium at 37 °C, individual diploid fusants were randomly picked for DNA extraction and genotyping. For each fusant, the *MAT***a** and *MAT*α -specific primer pairs at the *STE12***a** *and STE12α* genes were respectively used to confirm that the fusants were heterozygous at the mating type locus, following protocols described in Yan et al. [28]. The confirmed fusants were then genotyped at various loci in their mitochondrial genomes, as described below.

### Identification of mitochondrial genotypes

The mitochondrial genotypes of the parental strains and their diploid fusants were determined by PCR or PCR-restriction fragment length polymorphisms (PCR-RFLP) at the following marker gene loci: NADH dehydrogenase subunits #2, #4 and #5 (*ND2*, *ND4*, and *ND5*), *COB1*, *LsrRNA,* and *COX1*. The primers used for identifying mitochondrial genotypes are listed in Table 2. The mitochondrial genotypes at *ND2* and *ND4* loci were determined based on PCR-RFLP as described previously [15, 25]. The genotypes at loci *ND5*, *COX1, COB1* and *LsrRNA* were determined by PCR using primers located in intron-flanking regions as described by Toffaletti et al. [26] and Litter et al. [14]. Because the parental strains (YZX2 and the four JEC21 derivatives CHY618, CHY620, CHY647, and CHY648) did not differ at *COB1* or *LsrRNA* loci (see Results below), we only genotyped the diploid fusants from these crosses at the following four mitochondrial loci: *ND2*, *ND4*, *ND5* and *COX1*.

**Table 2:** Primers for amplifying mtDNA fragments for genotyping in this study

Primer pair name	Primer sequence (5’→3’)	Gene name and location of primer
ND2F	CAAGCTGCACCATTCCATA	*ND2*
ND2R	CCATTAGTGGTGGTACTCC	*ND2*
ND4F	GGGAGAATTTGATTCAAGTGCAAC	*ND4*
ND4R	CATACATGGAAAGGTACTAG	*ND4*
Da20	GACACTACACAAGATGCCTC	*COX1* exon 1
Da3	GCAATAGCATATACCATCCCG	*COX1* exon 3
Da22	CTCGAGCTTACTTTACAGCAG	*COX1* exon 3
Da19	GTACTACTCCTGTTAGTCCTC	*COX1* exon 4
Da26	CAACGGCATACGGTGGATCTATCC	*COX1* exon 4
Da15	CTGTTAGATATGATGGTGTGC	*COX1* exon 6
COB1F	CCACAACCTATTAACATTAGCTACGC	*COB1* exon 1
COB1R	CGTCTCCATCTACAAAGCCAGCAAAC	*COB1* Intron 2
ND5F	CTATTGGTGTTACAGGAGCTCAC	*ND5*
ND5R	GAGCCTTCATACCTGCCTTATTTGC	*ND5*
LsrRNAF	CAGCAGAACCCTTCCCAGC	Upstream *LsrRNA*
LsrRNAR	CCTCCACTGTCTCATGCGG	*LsrRNA* exon 3
Cox1F	TGTCTGGAGCTGGTAACCAAT	*COX1* exon 1
Cox1R	AAGAGGTGTTCATATAGAACTGG	*COX1* exon 1
ATP6F	GACACACTTTATTACATCTCCAC	*ATP6*
ATP6R	GAAGTTCAATGGCATCCTTG	*ATP6*
N2A8F	AACTCCCCACATAGTTATGG	*ND2-ATP8ig* ^a^
N2A8R	CATCCCTGTTATTAATTCACT	*ND2-ATP8 ig*
ATP8F	TTTCAATGGGTGCTGTGTTC	*ATP8-COX1ig* ^b^
ATP8R	CCGAATGTAATTTGGTTTACCC	*ATP8*
ATP9F	CGGACTATCAGGAGCTGGAG	*ATP9*
ATP9R	TTGGTGGTCACCGTTTAGAA	*ATP9-COX1ig* ^c^
ND6F	ACTTGATCTTCTTGCATTTGG	*ND6*
ND6R	TTATGTTCGTGGTCGTAGACA	*ND6*

^a^*ND2-ATP8ig* indicates that the primer is located in the intergenic region between *ND2* and *ATP8*

^b^*ATP8-COX1ig* indicates that the primer is located in the intergenic region between *ATP8* and *COX1*

^c^*ATP9-COX1ig* indicates that the primer is located in the intergenic region between *ATP9* and *COX1*

### DNA sequencing

Our results showed that the HEGs in *COX1* was mobile during sexual crosses (see Results below). To determine the border regions of HEG transpositions, we PCR-amplified the *COX1* gene together with its flanking sequence using the following primer pairs described in Table 2: Da20 and Da3; Da22 and Da19; Da26 and Da15; N2A8F and N2A8R; ATP8F and ATP8R; ATP9F and ATP9R; and Cox1F and Cox1R. PCR products were purified using a PCR cleaning kit (Qiagen). The purified PCR products were sent for sequencing using both the forward and reverse primers at the Mobix Laboratory at McMaster University.

## Results

### The distribution of mitochondrial HEGs and their associated introns among the five parental strains

Based on the published mitochondrial genome sequences, we synthesized primers to confirm the presence/absence of all mitochondrial introns in the five parental strains used in this study (Table 2). Table 3 summarized the distributions of introns in these five parental strains as well as two reference strains. Our analyses indicated that strains CHY618, CHY620, CHY647 and CHY648 had all the 10 introns described for the sequenced strains JEC21 and IFM5844, including five introns in the *COX1* gene, two each in the *COB1* and *LsrRNA* genes, and one in the *ND5* gene. This result is consistent with previous reports [14, 26] and with our expectation because JEC21 was the progenitor strain for these four strains.

**Table 3:** Mitochondrial intron distribution in the parental strains used in this study

	JEC21	CHY618	CHY620	CHY647	CHY648	YZX2	H99
*COB1* intron #1	+	+	+	+	+	+	–
*COB1* intron #2	+	+	+	+	+	+	+
*LsrRNA* intron #1	+	+	+	+	+	+	–
*LsrRNA* intron #2	+	+	+	+	+	+	–
*ND5* intron	+	+	+	+	+	–	–
*COX1* intron #1	+	+	+	+	+	–	–
*COX1* intron #2	+	+	+	+	+	–	–
*COX1* intron #3	+	+	+	+	+	+	–
*COX1* intron #4	+	+	+	+	+	–	–
*COX1* intron #5	+	+	+	+	+	–	–

Their distributions in the two common lab strains JEC21 and H99 are also described here

+, presence of the intron; −, absence of the intron

**Fig. 1:**
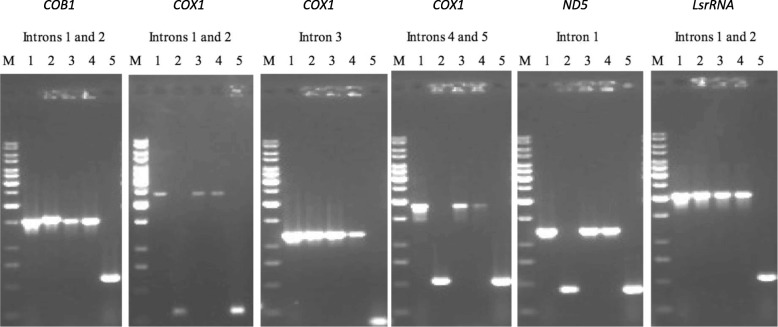
The distribution of introns among strains of *C. neoformans*. In total there are 10 introns: two within the *COB1* gene, five within the *COX1* gene, one within the *ND5* gene, and two within the *LsrRNA* gene. These fragments are amplified by primers flanking these introns. Lanes 1, 2, 3, 4 and 5 represent strains JEC21, YZX2, CHY618, CHY620 and H99 respectively. JEC21 is the model serotype D strain and H99 is the model serotype A strain. JEC21 and H99 are used here as intron positive and negative controls respectively and our results here confirmed the original description by Litter et al. (2005) and Toffalentii et al. (2004). Lane M is the marker 1 kb ladder. As can be seen from the gels, the *MAT***a** parental strain YZX2 differs from the *MAT*α strains CHY618 and CHY620 at five introns: one located within *ND5* and four located within the *COX1* gene (Introns 1, 2, 4, and 5)

In contrast, the mitochondrial genome of the *MAT***a** parental strain YZX2 for our crosses was found to contain five introns with two each within the *COB1* and *LsrRNA* genes and one within the *COX1* gene. The intron distribution in YZX2 is thus different from those of the two published serotype A strains (1 intron only in serotype A strains H99 and IFO410, refs #14 and 26) as well as the two published serotype D strains (10 introns in serotype D strains, JEC21 and IFM5844) (Fig. 1). Thus, in total, five introns absent in the mitochondrial genome of YZX2 but present in the other four strains in Table 1 (i.e. CHY618, CHY620, CHY647 and CHY648) can be used for genotyping intron mobility in this study: one located within the *ND5* gene and four located within the *COX1* gene. Sequence analysis showed that the *COX1* intron in strain YZX2 corresponded to intron #3 of the *COX1* gene in the other four strains used in this study (Table 3). Among the four polymorphic introns within the *COX1* gene, three contained HEGs (introns #1, #2 and #4). Together, the differences in intron distribution between the *MAT***a** YZX2 strain and the four *MAT*α strains shown in Table 1 provided us an opportunity to examine the potential mobility of introns in *ND5* and *COX1* genes during sexual crosses.

### The HEG-containing introns in the *COX1* gene are mobile during sexual mating

Before examining intron distribution among progeny populations of the four crosses, we first investigated whether all the fusants selected based on auxotrophic and NAT/NEO-resistance markers contained mating type alleles from both parental strains in all four crosses. Our analyses confirmed that all selected fusants were heterozygous at the mating type locus, representing true mating products. For each cross, we then used gene-specific PCR-RFLP markers at the *ND2* and *ND4* loci and PCR fragment length markers flanking introns in genes *ND5* and *COX1* to determine the mitochondrial genotype of each diploid fusant. The summary results for all four crosses are presented in Table 4.

**Table 4:** Progeny mitochondrial genotypes and the number of progeny in each genotype class from four crosses

Cross	CaNa^a^	CαNα^b^	CαNa^c^	CaNα^d^	CαNr^e^	CbNb^f^	Total
1. CHY618 X YZX2 (*MAT****a***)	5	70	30	0	3	8	116
2. CHY620 X YZX2 (*MAT****a***)	254	12	2	1	0	4	273
3. CHY648 X YZX2 (*MAT****a***)	120	6	1	0	0	1	128
4. CHY647 X YZX2 (*MAT****a***)	4	43	30	0	3	11	91

^a^, CaNa refers to diploid fusants having both the *COX1* allele and other genetic markers originating from the *MAT***a** parent YZX2

^b^, CαNα refers to a diploid fusant having both the HEG-containing introns in *COX1* gene and other genetic markers originating from the *MAT*α parent

^c^, CαNa refers to diploid fusants having the HEG-containing introns in *COX1* originating from the *MAT*α parent but other genetic markers originating from the *MAT***a** parent YZX2

^d^, CaNα refers to diploid fusants having the *COX1* allele originating from the *MAT***a** parent but other genetic markers originating from the *MAT*α parent

^e^, CαNr refers to diploid fusants having the HEG-containing introns in *COX1* originating from the *MAT*α parent and recombinant mtDNA genotype with *ND2* and *ND5* alleles from the *MAT***a** parent and *ND4* from the *MAT*α parent

^f^, CbNb refers to diploid fusants having alleles from both parents at all analyzed loci

Of the four crosses, we first analyzed diploid fusants from the cross between strains CHY618 (*MAT*α Δsxi1*α*) and YZX2 (*MAT***a**). Similar to results from a previous study [25], fusants from the CHY618 (*MAT*α Δsxi1*α*) x YZX2 (*MAT***a**) cross (cross #1, Table 4) showed a diversity of mitochondrial genotypes. Indeed, significantly more progeny from this cross inherited the mitochondrial genome from the *MAT*α parent than from the *MAT***a** parent (Chi-square statistic = 33.86; df = 1; *p* = 0, fusants with recombinant and heteroplasmic mtDNA genotypes were excluded in this test). Furthermore, there was a higher frequency of heteroplasmic or recombinant mtDNA genomes (recombinant genotype based only on alleles at *ND2*, *ND4* and *ND5* genes) than those of its reference cross with the wild-type *SXI1*α gene CHY620 x YZX2 (cross #2 in Table 4) (Chi-square statistic = 12.0839; df = 1; *p* = 0.000509). Interestingly, when we examined the progeny genotypes at the *COX1* gene locus*,* 95.2% of the diploid fusants (100/105; excluding the 11 recombinant or heteroplasmic fusants) inherited the HEG-containing introns in the *COX1* gene from the *MAT*α parent in the CHY618 x YZX2 cross (cross #1, Table 4), significantly more than the alleles at the *ND2, ND4* and *ND5* loci (~ 75%) (Chi-square statistic = 16.1345; df = 1; *p* = 0.000059; the 11 recombinant or heteroplasmic fusants were excluded from this comparison). The result clearly indicates that HEG-associated introns in *COX1* gene are preferentially inherited by progeny from this cross.

Specifically, in the CHY618 (*MAT*α Δsxi1*α*) x YZX2 (*MAT***a**) cross (cross #1, Table 4), 30 (of 116) diploid fusants inherited the intronless mitochondrial markers *ND2*, *ND4* and *ND5* from their *MAT***a** parent YZX2 but contained the HEG-containing *COX1* introns from the *MAT*α parent CHY618 (*MAT*α Δsxi1*α*). This transmission pattern in favor of the HEG-containing *COX1* introns over the intronless markers at *ND2*, *ND4*, and *ND5* is significantly more frequent than that found in the control cross CHY620 x YZX2 (cross #2 in Table 4) where only two of the 273 diploid fusants had such a recombinant mitochondrial genotype (Chi-square statistic = 68.09; df = 1; *p* = 0). Furthermore, there was no evidence of reciprocal recombinants among progeny from the CHY618 x YZX2 cross that contained the mtDNA markers *ND2*, *ND4*, and *ND5* from the *MAT*α parent but without any of the four HEG-containing introns in the *COX1* gene from YZX2 (Table 4). The results from crosses #1 and #2 clearly indicate that the deletion of *SXI1*α was associated with the spread of the HEG-associated introns in the *COX1* gene.

Among the 30 progeny that inherited HEG-associated introns independent of the majority of their mitochondrial genomes, most (29/30) contained all four introns in the *COX1* gene from the MATα parent. One progeny inherited only two of the four introns (introns #1 and #2 but not #4 and #5) in the *COX1* gene. (Both parental strains already have intron #3.)

Unlike the inheritance pattern of HEG-containing introns in the *COX1* gene, the transmission of the *ND5* intron followed the same inheritance pattern as the intronless genetic markers in the *ND2* and *ND4* genes. This result suggests that the intron in *ND5* is not mobile. This observation is consistent with our expectation as the intron in *ND5* gene of strain CHY618 contained no LAGLIDADG motif (i.e. no HEG), and thus is not expected to move, unlike the HEG-containing introns in the *COX1* gene.

### The spread of the HEG-associated introns is confined to the *COX1* gene region

**Fig. 2:**
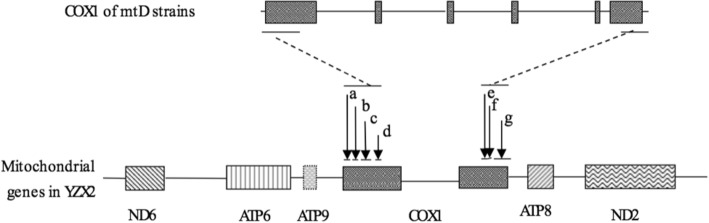
Schematic representation of the mitochondrial genomic region surrounding *COX1* and the borders of intron homing in 10 of the 30 diploid fusants with the CαNa mitochondrial genotypes. The arrows show the regions of integration borders. All the borders are located either within the *COX1* gene or within the intergenic region between *COX1* and two other genes (between *ATP9* and *COX1* for the left border and between *COX1* and *ATP8* for the right border), close to *COX1* translation initiation and termination sites. Arrow “a” shows the left borders of two strains (OYZ1 and YZ151); arrow “b”, the left borders of two strains (YZ150 and YZ153); arrow “c”, the left borders of two strains (OYZ2 and YZ159); and arrow “d”, the left borders of four strains (YZ152, YZ155, YZ158 and YZ160). Arrow “e” points to the right borders of two strains (YZ159 and YZ160); arrow “f”, the right border of strain YZ158; arrow “g”, the right border of the remaining seven strains (OYZ1, OYZ2, YZ150, YZ151, YZ152, YZ153 and YZ155)

To further examine the extent of the transposed regions within and around the *COX1* gene, we sequenced the *COX1* gene and its flanking regions for 10 random fusants (out of 30 mentioned above from the cross CHY618 x YZX2) that contained the transferred introns in *COX1* as identified using the 6 pairs of primers listed in Table 2. Sequences from these fusants were then compared to sequences from the two parental strains. Our comparisons showed that the recombination borders for all 10 mating fusants lie close to the 5′ and 3′ ends of the *COX1* gene, either within the coding region or in the adjacent flanking regions less than 48 base pairs from the translation start or end sites of the *COX1* gene (Table 5, Fig. 2). In contrast, as mentioned previously, the three genes *ND2, ND4* and *ND5* all inherited their alleles from the *MAT***a** parent YZX2 and showed no evidence of recombination among them within this sub-population of fusants (Table 4). In addition, these three genes are widely spaced in the *C. neoformans* mitochondrial genome [14, 26]. Furthermore, the frequency of HEG-associated intron transposition (30/35) is significantly higher than that of recombination based on the three gene markers *ND2, ND4* and *ND5* (3/38, Table 2) (Chi-square statistic = 44.54; df = 1; *p* = 0)*.* Taken together, the high frequency and high specificity of HEG-associated intron transpositions within the *COX1* gene and the lack of evidence for recombination among the other three markers in these 30 fusants suggest that the 30 novel mitochondrial genotypes were generated via homing of HEG-containing introns in the *COX1* gene, and not due to mitochondrial recombination.

**Table 5:** Locations of regions containing recombination borders in 10 diploid fusants from cross CHY618 (Δ*sxi1*α) x YZX2

Strains	Left border within this region	Location of the left border	Right border within this region	Location of the right border
OYZ1	13,127~ 13237^a^	*-COX1* ^b^	14,440~ 14,786	*COX1-* ^c^
OYZ2	13,639~ 13,840	*COX1* ^d^	14,440~ 14,786	*COX1-*
YZ150	13,456~ 13,588	*COX1*	14,440~ 14,786	*COX1-*
YZ151	13,127~ 13,237	*-COX1*	14,440~ 14,786	*COX1-*
YZ152	13,855~ 13,887	*COX1*	14,440~ 14,786	*COX1-*
YZ153	13,456~ 13,588	*COX1*	14,440~ 14,786	*COX1-*
YZ155	13,855~ 13,887	*COX1*	14,440~ 14,786	*COX1-*
YZ158	13,855~ 13,887	*COX1*	14,374~ 14,407	*COX1*
YZ159	13,639~ 13,840	*COX1*	14,297~ 14,374	*COX1*
YZ160	13,855~ 13,887	*COX1*	14,297~ 14,374	*COX1*

^a^The numbers here show the location of recombination borders using the published serotype A mitochondrial genome (NC_004336) as a reference

^b^*-COX1* indicates that the left border of recombination is located either within the *COX1* gene or in the intergenic region between *ATP9* and *COX1*

^c^*COX1-* indicates that the right border of recombination is located either within the *COX1* gene or in the intergenic region between *COX1* and *ATP8*

^d^*COX1* indicates that the border of recombination is located within the *COX1* gene

### *SXI1*α gene inhibits the spread of HEG-associated introns in *COX1*

If the hypothesis that uniparental organelle inheritance evolved to prevent the spread of selfish genetic elements were correct, we would expect any gene critical for uniparental mitochondrial inheritance, like *SXII*α in this study, to play a role in the transmission of selfish elements. To test whether *SXI1*α can limit the transmission of HEGs in the mitochondrial genome, we analyzed three more crosses. In contrast to the cross between CHY618 and YZX2 described above, transmission of HEG-associated introns was significantly inhibited in crosses involving functional *SXI1*α (crosses #2 and #3, Table 4). For example, in cross #2 between CHY620 and YZX2, 93.4% of the fusants (255/273) did not inherit the *COX1* HEGs from the *MAT*α parent CHY620 (Table 4). Of the remaining 18 fusants from this cross, 12 had mtDNA from only the *MAT*α parent CHY620; four had mtDNA from both parents at all loci (heteroplasmy); and two had most of the mtDNA from the *MAT***a** parent but contained the four additional *COX1* introns from the *MAT*α parent (Table 4). These last two novel mtDNA genotypes were likely derived from HEG-mediated intron homing, similar to that proposed for the 30 progeny from cross #1 described above.

Similar to cross #2, in cross #3 (Table 4), re-introducing the functional *SXI1*α allele back to CHY618 (Δ*sxi1α*) restored uniparental mitochondrial inheritance and consequently also limited the spread of HEG-containing introns among the fusants. Furthermore, the results in cross #4 showed that neither the plasmid vector pPM8 nor the selection markers influenced HEG-mediated intron transmission (Table 4, cross #4).

**Fig. 3:**
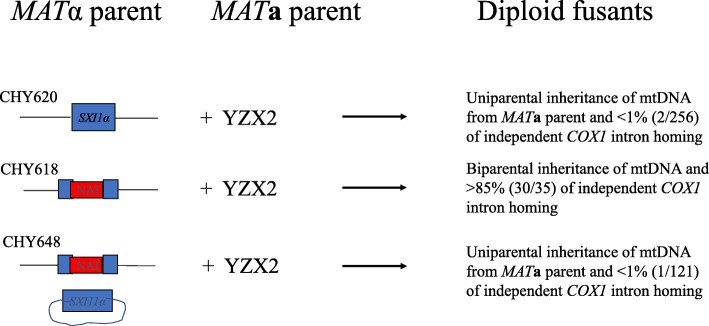
Summary of the effects of *SXI1*α on mitochondrial DNA inheritance and mobility of HEG-containing introns in *COX1* gene during sexual mating in *Cryptococcus neoformans.* The genotypes of parental strains are shown in Table 1

Together, our results from these four crosses clearly indicated that the presence of a functional *SXI1*α gene significantly inhibited the spread of HEG-associated introns from the intron-containing mitochondrial genome to the homologous but intronless sites in progeny mitochondrial genomes during sexual mating. The deletion of *sxi1α* led to a significantly greater frequency of HEG-containing introns in *COX1* gene than other genes in mating fusants. Together, our results suggest that *SXI1α* not only controls uniparental mitochondrial inheritance but also limits the spread of the HEG-containing introns in *C. neoformans* (Fig. 3)*.*

## Discussion

HEGs and their associated introns have been observed in organelle genomes of many groups of organisms including Fungi, Ameobozoa, Plantae, Chromalveolata, Rhizaria, Excavata, and Metazoa [29]. However, the motility of these introns and how their transmission is controlled remain little known. In this study, we examined the mobility of mitochondrial HEG-containing introns in the human pathogenic yeast *C. neoformans*. We found that while the intron in the *ND5* gene was not mobile, the four HEG-associated introns in the *COX1* gene were mobile. Furthermore, we demonstrated that the *MAT*α-specific homeodomain gene *SXI1*α influenced the mobility of the HEG-associated introns. Deletion of this gene not only disrupted uniparental mitochondrial inheritance but also enabled the mobility of the HEG-containing introns in *COX1*.

### Recombination and unidirectional transfer of HEG-containing introns in mitochondria

In our crosses, we observed both homologous recombination and unidirectional transfer of HEG-containing introns in the mitochondrial genomes. These two processes have been observed in other species. For example, homologous mitochondrial genome recombination has been reported in the basidiomycete mushrooms *Coprinus cinereus* [30], *Agrocybe aegerita* [31] and *Pleurotus ostreatus* [32]. Similarly, independent transfers of HEG-associated introns have also been reported in other species including the baker’s yeast *Saccharomyces cerevisiae* [7], the plant fungal pathogen *U. maydis* [20, 21] and the unicellular algae *Chlamydomonas* species [9]. In species of the model filamentous fungal genus *Neurospora*, mitochondrial plasmids with sequences very similar to group I introns have been found capable of independent transfer to strains without such plasmids [33]. However, the control for the above-mentioned intron transfers between organelles remains unknown.

### Relevance of mitochondrial intron mobility to the evolution of uniparental mtDNA inheritance

In the great majority of sexual eukaryotes, mitochondria are inherited almost exclusively from a single parent [9, 11, 34]. Because uniparentally inherited genomes are prone to mutation accumulation [35–37], the predominance of uniparental mitochondrial inheritance has posed a serious challenge to evolutionary biologists. Indeed, many hypotheses have been proposed to explain the prevalence of uniparental mitochondrial inheritance [10, 36, 38, 39]. One commonly discussed hypothesis stated that uniparental organelle inheritance evolved to prevent the spread of selfish cytoplasmic DNA such as HEG-containing introns [10, 17–19]. However, experimental evidence for this hypothesis has been lacking.

In this study, we found that the sex-determining nuclear gene *SXI1*α was not only critical for ensuring uniparental mitochondrial inheritance, as was demonstrated previously [25], but also inhibited the spread of HEGs in the mitochondrial genome in *C. neoformans*. In the corn fungal pathogen, *U. maydis*, a nuclear gene *LGA2* was found to play a critical role in ensuring uniparental mitochondrial inheritance and in HEG transmission [20, 21]. Specifically, the absence of *LGA2* led to an increased number of progeny having an HEG-containing intron in the gene coding for the mitochondrial large subunit of ribosomal RNA (*LsrRNA*). However, unlike in our study, intronless markers independent of the HEG-containing intron in the *LsrRNA* gene were not investigated in their crosses. As a result, they were unable to determine whether the increased prevalence of the mobile intron was due to the loss of control for uniparental mitochondrial inheritance or due to enhanced intron mobility. In contrast, our data clearly showed that the absence of a functional *sxi1α* enhanced the spread of HEG-containing introns, independent of mitochondrial recombination and on top of biparental mitochondrial inheritance.

Our results also differ from those observed in the green algae *Chlamydomonas*. In *Chlamydomonas*, there are two types of organelles, the chloroplasts and the mitochondria. HEG transmission in the chloroplast genome of *Chlamydomonas* appears to be different from that in the mitochondrial genome. For HEGs in the chloroplast genome, either parent (*mt*- or *mt*+) can transmit HEG to sexual progeny [22, 23]. In contrast, in the mitochondrial genome, only the *mt*- parent transmits its mitochondrial HEG to zygotes [40]. The different HEG transmission patterns in these two organelle genomes suggest uniparental inheritance and intron mobility of these two organelle genomes are controlled by different genes and likely evolved independently. Nevertheless, data from this study and those found in *U. maydis* and *Chlamydomonas* all showed that uniparental mitochondrial inheritance can limit the spread of HEG-containing introns. However, only in *C. neoformans* has it been demonstrated that the absence of a mating type-specific gene *sxi1α* led to the over-transmission of HEG-containing introns independent of other mitochondrial genes.

While HEG-containing introns are among the most frequently discussed selfish genetic elements, there are also other types of deleterious cytoplasmic elements including intracellular parasites as well as defective organelle genes and genomes. These genetic elements may have a replication and/or transmission advantage relative to other genes in the host cell, resulting in their over-representation in subsequent generations that may cause deleterious effects for the hosts. For example, the petite phenotype in the baker’s yeast *S. cerevisiae* is typically characterized by large deletions in the mitochondrial genome and by impaired respiration [41]. In heteroplasmic cells containing both the wild-type mitochondrial genome and the petite mitochondrial genome with large deletions, the smaller mitochondrial genomes from petite mutants may exhibit a two-fold transmission advantage compared to the wild type mitochondrial genome [42]. While natural selection could eventually purge host cells with the defective mitochondrial genomes from the population, having a mechanism to prevent their transmission during sexual crosses could be highly beneficial.

## Conclusions and perspectives

In this study, we identified that the mating-type *α*-specific, sex-determining gene *SXI1*α could prevent the spread of HEG-containing introns in the mitochondrial genome in *C. neoformans* (Fig. 3)*.* However, we urge caution in extending our laboratory observations to natural populations. For technical reasons (i.e. crosses #1 and #4 are unable to produce dikaryotic hyphae, the typical mating products in CNSC, even in successful mating) and for comparative purposes among crosses, only diploid fusants were selected for analyses in this study. In contrast, mating in natural environments will likely produce dikaryotic hyphae and not diploid yeast cells. The distributions and interactions between parental mitochondria in cytoplasm could differ between dikaryotic hyphae and diploid yeast fusants. Furthermore, though diploid fusants have been reported in *C. neoformans* [28, 43], the genetic stability of the diploid fusants is currently not known. Further analyses of the distributions of HEG-containing introns among environmental and clinical strains are needed in order to understand how HEG-containing introns may have spread in nature.

*SXI1*α is a transcription factor and can influence the expressions of many genes [44]. Previous studies have demonstrated that *MAT*α is the dominant mating type in both environmental and clinical populations of *C. neoformans* and that strain JEC21 with the *MAT*α mating type is more virulent than its isogenic *MAT***a** partner JEC20 [28, 43]. In *C. neoformans*, the mating type loci are relatively complex, ~ 100 kb in length and encoding ~ 20 or more genes at each of the *MAT***a** and *MAT*α loci. The *SXI1*α gene is among the genes located within the *MAT*α locus. As a transcription factor, *SXI1*α likely controls the spread of HEG introns indirectly through regulating the expressions of other genes. Further investigations are needed in order to identify its downstream targets and the molecular processes involved in controlling intron homing.

Regardless of the molecular mechanisms governing intron mobility, homing endonucleases have shown significant potential in genome editing and are now used in the fields of agriculture and human health [45]. For example, because HEG can spread quickly in populations, HEGs have been tested for controlling the hosts of malaria parasites, mosquitoes [46]. In our study, four of the five introns in the *COX1* gene of strain CHY618 contained HEGs. Protein sequences of these HEGs in *C. neoformans* differ from other reported HEGs, with the best match to a homing endonuclease found in the mitochondrial genome of watermelon (71% amino acid sequence identity, uniprot B4XPH0). At present, we do not know which of the HEGs within the *COX1* gene initiated the intron homing process nor which HEG has the highest activity. Knockout or over-expression of each of the four HEGs separately might allow us to pinpoint the functional significance of individual HEGs in *COX1* and their recognition sequences [45, 47]. Such knowledge could help us understand the molecular processes of intron transmission in fungi, their potential roles in fungal pathogenesis, and their putative applications in genome editing.

## Abbreviations

*MAT*α: Mating type α
CNSC: *Cryptococcus neoformans* species complex
*COB1*: Cytochrome b
*COX1*: Cytochrome c oxidase I
HEG: Homing endonuclease gene
*LsrRNA*: The large subunit of the ribosomal rRNA gene
MAT: Mating type
*MAT***a**: Mating type **a**
*ND2*: NADH dehydrogenase subunit 2
*ND4*: NADH dehydrogenase subunit 4
*ND5*: NADH dehydrogenase subunit 5
PCR-RFLP: Polymerase chain reaction – restriction fragment length polymorphisms
SD: Synthetic dextrose medium
YEPD: Yeast extract peptone dextrose medium
